# Supplementary material for: Trends and Determinants of Operative Vaginal Delivery at Two Academic Hospitals in Johannesburg, South Africa 2005–2019
Source: Int J Environ Res Public Health. 2022 Dec 3;19(23):16182. doi: 10.3390/ijerph192316182 (PMC9735469; doi:10.3390/ijerph192316182)
Supplement: Supplementary file 1 [file ijerph-19-16182-s001.zip › ijerph-1912785-supplementary.pdf]

Trends and determinants of operative vaginal delivery at two academic hospitals in Johannesburg, South Africa. 2005–2019

Afikile Dutywa[1], Gbenga Olorunfemi[2], Langanani Mbodi[1]

[1]Department of Obstetrics and Gynaecology, School of Clinical Medicine, Faculty of Health Science, University of the Witwatersrand, Johannesburg 2000, South Africa

[2]Division of Epidemiology and Biostatistics, School of Public Health, University of the Witwatersrand, Johannesburg 2000, South Africa

Correspondence to Dr Afikile Dutywa, School of clinical medicine, University of Witwatersrand, Parkton, Johannesburg

Email: dra.dutywa@gmail.com

Running title: Trends in operative vaginal delivery in Johannesburg

Supplementary Tables.

Supplementary Table S1. Trends in overall mode of delivery at both hospitals

	Overall				
**Year**	**Total Birth**	**Caesarean section(C/s)**	**Caesarean section rate (%)**	**OVD**	**Ovd rate (%)**
2005					
2006					
2007					
2008					
2009	29600	10521	76,19	377	2,662
2010	28158	10558	79,85	370	3.01
2011	31338	12112	84,52	478	3,58
2012	29741	15524	87,52	560	3,62
2013	30472	11892	84,62	597	4,16
2014	30158	12387	86,9	564	3,88
2015	24525	9940	86,52	412	2,87
2016	23406	9845	86,52	412	2,87
2017	27865	12234	93,1	299	2,02
2018	27695	12115	89,26	313	2,31
2019	24517	12807	118,27	278	2,56
OVD: Operative vaginal delivery					

Supplementary Table S2. Trends in mode of delivery at the two academic hospitals at Johannesburg, South Africa

	CHBAH					CMJAH				
Year	Total Birth	C/S	C/S rate (%)	OVD	Ovd rate (%)	Total Birth	C/S	CS Rate (%)	OVD	Ovd rate (%)
2005	20318	5765	28.37	169	0.83					
2006	22826	6066	26.57	216	0.95					
2007	24286	6703	27.60	294	1.21					
2008	23197	7241	31.22	309	1.33					
2009	22825	7628	33.42	280	1.23	6775	2893	42.70	97	1.43
2010	21572	7628	35.36	273	1.27	6586	2930	44.49	97	1.47
2011	23406	8179	34.94	294	1.26	7932	3933	49.58	184	2.32
2012	22211	7994	35.99	434	1.95	7530	3880	51.53	126	1.67
2013	22847	8165	35.74	420	1.84	7625	3727	48.88	177	2.32
2014	22300	8587	38.51	400	1.79	7858	3800	48.39	164	2.09
2015	19059	7306	38.33	358	1.88	5466	2634	48.19	54	0.99
2016	19747	8024	40.63	310	1.57	3659	1821	49.77	36	0.98
2017	21223	8806	41.49	240	1.13	6642	3428	51.61	59	0.89
2018	19334	8196	42.39	211	1.09	8361	3919	46.87	102	1.22
2019	18466	8405	45.52	183	0.99	6051	4402	72.75	95	1.57
Total	323,617	114,693	536,98	4,391	20,32					
OVD: Operative vaginal delivery; C/S : Caesarean Section; CHBAH: Chris Hani Baragwanath Academic Hospital; CMJAH: Charlotte Maxeke Johannesburg Academic Hospital.										

Supplementary Table S3. Trends in type of operative vaginal deliveries at the two academic hospitals at Johannesburg (2005-2019)

		CHBAH						CMJAH						
Year	OVD	Forceps	Forceps rate (%)	*Forceps rates	Vacuum (number)	*Vacuum Rate (%)	Vacuum rate^	OVD	Forceps	Forceps rate (%)	*Forceps rate	Vacuum	Vacuum Rate (%)	*Vacuum Rate (%)
2005	169	69	40.83	0.35	100	59.17	0.51							
2006	216	41	18.98	0.19	175	81.02	0.79							
2007	294	69	23.47	0.29	250	85.03	1.06							
2008	309	39	12.62	0.17	270	87.38	1.20							
2009	280	20	7.14	0.09	260	92.86	1.14	97	17	17.53	0.25	80	82.47	1.20
2010	273	28	10.26	0.13	245	89.74	1.10	97	15	15.46	0.22	82	84.54	1.22
2011	294	33	11.22	0.15	261	88.78	1.15	184	70	38.04	0.89	114	61.96	1.45
2012	434	134	30.88	0.62	300	69.12	1.39	126	26	20.63	0.35	100	79.37	1.34
2013	420	51	12.14	0.23	369	87.86	1.66	177	20	11.30	0.27	157	88.70	2.08
2014	400	42	10.50	0.19	300	75.00	1.36	164	36	21.95	0.46	128	78.05	1.65
2015	358	96	26.82	0.51	262	73.18	1.38	54	14	25.93	0.26	40	74.07	0.74
2016	310	35	11.29	0.11	275	88.71	0.83	36	9	25.00	0.24	27	75.00	0.73
2017	240	40	16.67	0.19	200	83.33	0.97	59	18	30.51	0.27	41	69.49	0.62
2018	211	16	7.58	0.09	195	92.42	1.04	102	22	21.57	0.29	80	78.43	1.04
2019	183	14	7.65	0.08	169	92.35	0.92	95	17	17.89	0.19	78	82.11	0.88
Total														
*. Per 100 total births														

Supplementary Table S4. Comparison of the types of delivery at the two academic hospitals in Johannesburg

**Hospital**	**Vacuum**	**Wrigley's Forceps**	**Kielland's forceps**	**Vacuum and Forceps**	**Normal vaginal delivery**	**Total**	**P-value**
CHBAH	83(50.00)	8(80.00)	1(100.00)	0(0.00)	92(51.40)	184(51.40)	0.140
CMJAH	83(50.00)	2(20.00)	0 (0.00)	2(100.00)	87(48.60)	174(48.60)	

CBAH: Chris Hani Baragwanath Academic Hospital; CMJAH: Charlotte Maxeke Johannesburg Academic Hospital
